# Supplementary material for: Mindset and Reflection—How to Sustainably Improve Intra- and Interpersonal Competences in Medical Education
Source: Healthcare (Basel). 2023 Mar 14;11(6):859. doi: 10.3390/healthcare11060859 (PMC10048539; doi:10.3390/healthcare11060859)
Supplement: Supplementary file 1 [file healthcare-11-00859-s001.zip › File S3-Interviewees'_Demographics.pdf]

Demographics of interview partners

Sampling category	Profession	Sex / Age	Professional years	Background
Medical professional – Physician – high valuation of IICs	Physician<br>Medical teacher	Male / 58 years	22 years	• Teacher for patient centred communication<br>• Working on medical curricula<br>• Clinical mediator in residency programme
Medical professional – Physician – high valuation of IICs	Physician<br>Medical teacher	Female	26 years	• General Medicine<br>• Master of medical education<br>• Educational research<br>• Training representative for physicians' training focused on communicative competences
Medical professional – Physician – high valuation of IICs	Physician<br>Medical teacher	Male / 57 years	33 years	• Chair of the department of psychosomatic medicine
Medical professional – Physician – high valuation of IICs	Physician<br>Medical teacher	Male / 63 years	38 years	• General physician<br>• Communication skills trainer
Medical professional – Physician – high valuation of IICs	Physician<br>Medical teacher	Female / 37 years	4 years	• General physician<br>• Communication skills trainer<br>• PhD palliative care
Medical professional – Physician – high valuation of IICs	Physician<br>Psychological teacher	Female / 59 years	> 30 years	• Teacher for clinical psychology and psychotherapy
Medical professional – Physician – high valuation of IICs	Physician	Female / 32 years	4 years	• Assistant physician for neurology and psychiatry
Medical professional – Physician – restrictive valuation of IICs	Physician<br>Medical teacher	Male / 51 years	24 years	• Chair of the department for cardiology<br>• Deputy hospital director
Medical professional – Physician – restrictive valuation of IICs	Physician	Male / 33 years	3 years	• Assistant physician orthopaedics / trauma surgery
Medical profession –	Physician<br>Patients' advisor	Female / 60 years	34 years	• Medical advisor for patients in health insurance

patients' representation				
Medical profession – medical students	Medical student	Male / 27 years	-	• 6th year-medical student
Medical profession – medical students	Medical student	Female / 23 years	-	• 4th year-medical student
Medical profession – nursing	Medical teacher	Female / 57 years	31 years	• Nurse<br>• Trainer palliative care<br>• Therapy research
Medical profession – nursing	Nurse	Female / 51 years	33 years	• Nurse<br>• Quality management
Other profession with professional view on medical culture	Health journalist	Female	33 years	
Other profession with professional view on medical culture – patients' representation	Pedagogue Patients' representative	Male / 57 years	31 Years	• Patients' representative at "Federal centre for health education" (BzgA)
Other profession with professional view on medical culture – patients' representation	Patients‘ advisor	Male / 38 years	14 years	• Health economy<br>• Health insurance management<br>• Management of "unabhängige Patienten Beratung Deutschland", an independent patient counseling center in Germany
Other profession with professional view on medical culture	Social worker Medical teacher	Female / 61 years	36 years	• Teaching and researching at department of nursing for intrapersonal skills, interpersonal communication and domestic violence
Other profession with professional view on medical culture	Pedagogue	Female / 53 years	20 years	• Teaching<br>• Training representative of teachers' training
Other profession with professional view on medical culture	Pilots‘ trainer	Male / 61 years	35 years	• Pilot<br>• Aviation Trainer<br>• Researcher for aviation security

Other profession with professional view on medical culture	Lawyer	Male / 70 years	46 years	• Legal studies<br>• Management consulting
