# Supplementary figures and images for: Mindset and Reflection—How to Sustainably Improve Intra- and Interpersonal Competences in Medical Education
Source: Healthcare (Basel). 2023 Mar 14;11(6):859. doi: 10.3390/healthcare11060859 (PMC10048539; doi:10.3390/healthcare11060859)

Steps of coding

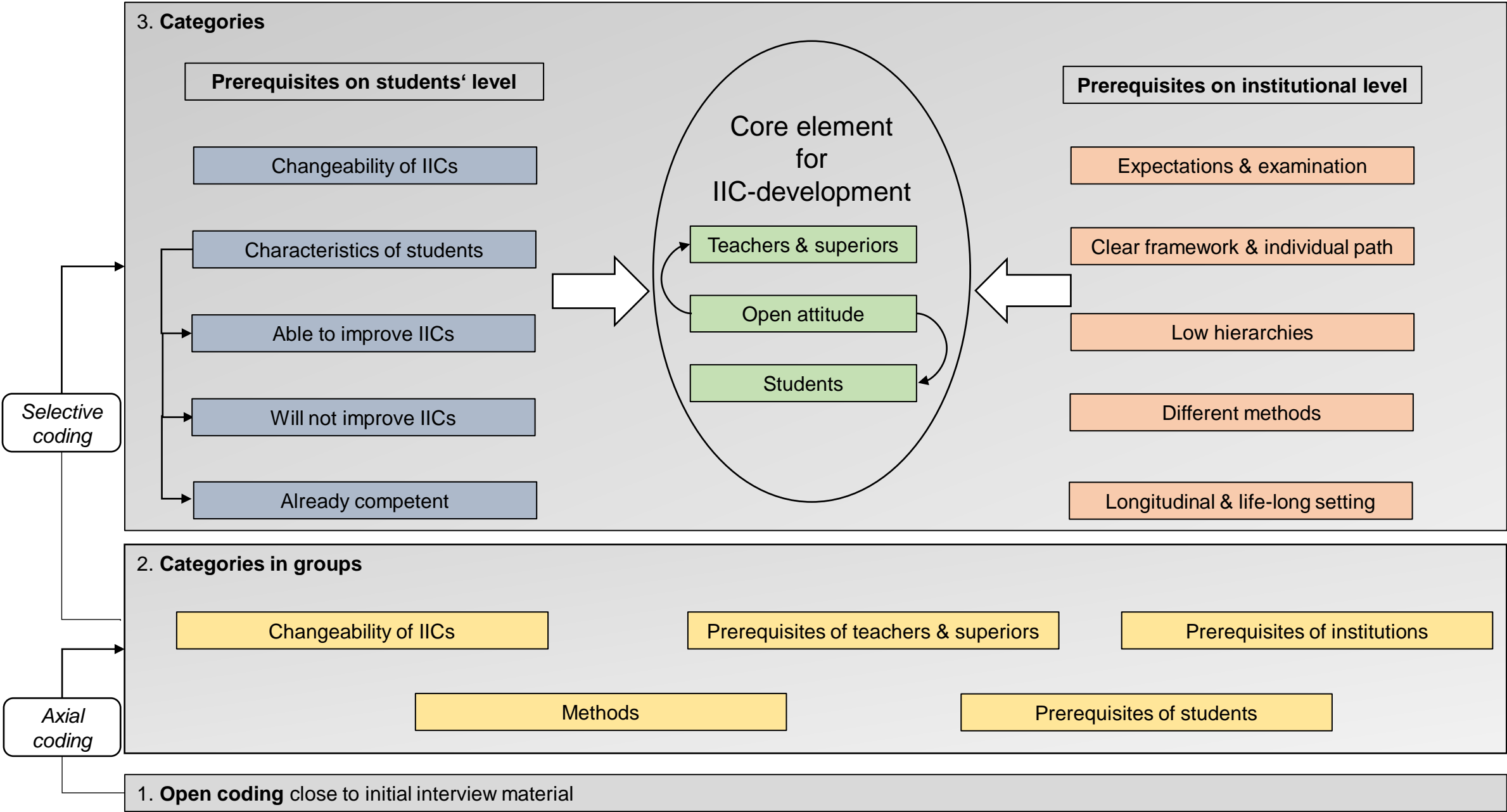

Supplement: Supplementary file 1 [file healthcare-11-00859-s001.zip › File S4-Steps_of_coding.pdf]
